# The Paternal Landscape along the Bight of Benin – Testing Regional Representativeness of West-African Population Samples Using Y-Chromosomal Markers

**DOI:** 10.1371/journal.pone.0141510

**Published:** 2015-11-06

**Authors:** Maarten H. D. Larmuseau, Andrea Vessi, Mark A. Jobling, Anneleen Van Geystelen, Giuseppina Primativo, Gianfranco Biondi, Cristina Martínez-Labarga, Claudio Ottoni, Ronny Decorte, Olga Rickards

**Affiliations:** 1 KU Leuven, Forensic Biomedical Sciences, Department of Imaging & Pathology, Leuven, Belgium; 2 KU Leuven, Laboratory of Socioecology and Social Evolution, Department of Biology, Leuven, Belgium; 3 University of Leicester, Department of Genetics, Leicester, United Kingdom; 4 University of Rome Tor Vergata, Department of Biology, Rome, Italy; 5 University of L’Aquila, Department of Clinical Medicine, Public Health, Life and Environment, L’Aquila, Italy; 6 KU Leuven, Centre for Archaeological Sciences, Department of Earth and Environmental Sciences, Leuven, Belgium; 7 UZ Leuven, Laboratory of Forensic Genetics and Molecular Archaeology, Leuven, Belgium; Erasmus University Medical Center, NETHERLANDS

## Abstract

Patterns of genetic variation in human populations across the African continent are still not well studied in comparison with Eurasia and America, despite the high genetic and cultural diversity among African populations. In population and forensic genetic studies a single sample is often used to represent a complete African region. In such a scenario, inappropriate sampling strategies and/or the use of local, isolated populations may bias interpretations and pose questions of representativeness at a macrogeographic-scale. The non-recombining region of the Y-chromosome (NRY) has great potential to reveal the regional representation of a sample due to its powerful phylogeographic information content. An area poorly characterized for Y-chromosomal data is the West-African region along the Bight of Benin, despite its important history in the trans-Atlantic slave trade and its large number of ethnic groups, languages and lifestyles. In this study, Y-chromosomal haplotypes from four Beninese populations were determined and a global meta-analysis with available Y-SNP and Y-STR data from populations along the Bight of Benin and surrounding areas was performed. A thorough methodology was developed allowing comparison of population samples using Y-chromosomal lineage data based on different Y-SNP panels and phylogenies. Geographic proximity turned out to be the best predictor of genetic affinity between populations along the Bight of Benin. Nevertheless, based on Y-chromosomal data from the literature two population samples differed strongly from others from the same or neighbouring areas and are not regionally representative within large-scale studies. Furthermore, the analysis of the HapMap sample YRI of a Yoruban population from South-western Nigeria based on Y-SNPs and Y-STR data showed for the first time its regional representativeness, a result which is important for standard population and forensic genetic applications using the YRI sample. Therefore, the uniquely and powerful geographical information carried by the Y-chromosome makes it an important locus to test the representativeness of a certain sample even in the genomic era, especially in poorly investigated areas like Africa.

## Introduction

Human populations in the African continent exhibit a high linguistic, cultural, genetic and phenotypic diversity. More than 2000 distinct ethno-linguistic groups practice a wide range of subsistence patterns including agriculture, pastoralism and hunting-gathering [1, 2]. The genetic diversity in African populations is higher than in non-African populations due to the recent African origin of modern humans, yet it is still poorly studied in comparison with populations of Eurasia and the Americas [3]. Such a multifaceted and fragmented pattern of variation, often amplified by the presence of clans or other substructures within a local population, can lead to issues of representativeness of a single sample included for a specific African region in macro-geographic population genetic and forensic studies. Anthropologists have already acknowledged problems with overly simplistic interpretations of genetic variation patterns at a continental scale in Africa, and called for more regional and local studies, and for verification of the regional representativeness of specific population samples before using them for large-scale population and forensic genetic studies [4].

A locus of particular interest for recognizing the regional representativeness of a specific sample is the non-recombining region of the Y-chromosome, because it carries uniquely powerful geographical information [5, 6]. This locus has been used in interdisciplinary studies on human evolution and population genetics [7, 8], historic demography [9], behavioural studies [10, 11], forensic genetics [12] and genetic genealogy [13], often in combination with the mitochondrial genome, which can be equally or even more appropriate to explore population stratification on continental scales [14]. Despite its potential, Y-chromosomal variation has been poorly explored in Africa in comparison with Eurasia or America [15–17]. Most African Y-chromosomal studies have been done in Bantu-speaking populations to understand the expansion of Bantu languages [18, 19]. Nevertheless, the recent discovery of the lineage A00 within a Bantu-population in North Cameroon, which introduced an extremely ancient root to the Y-chromosomal phylogenetic tree, testifies to the still limited knowledge of Y-chromosomal variation in Africa [20].

**Fig 1:**
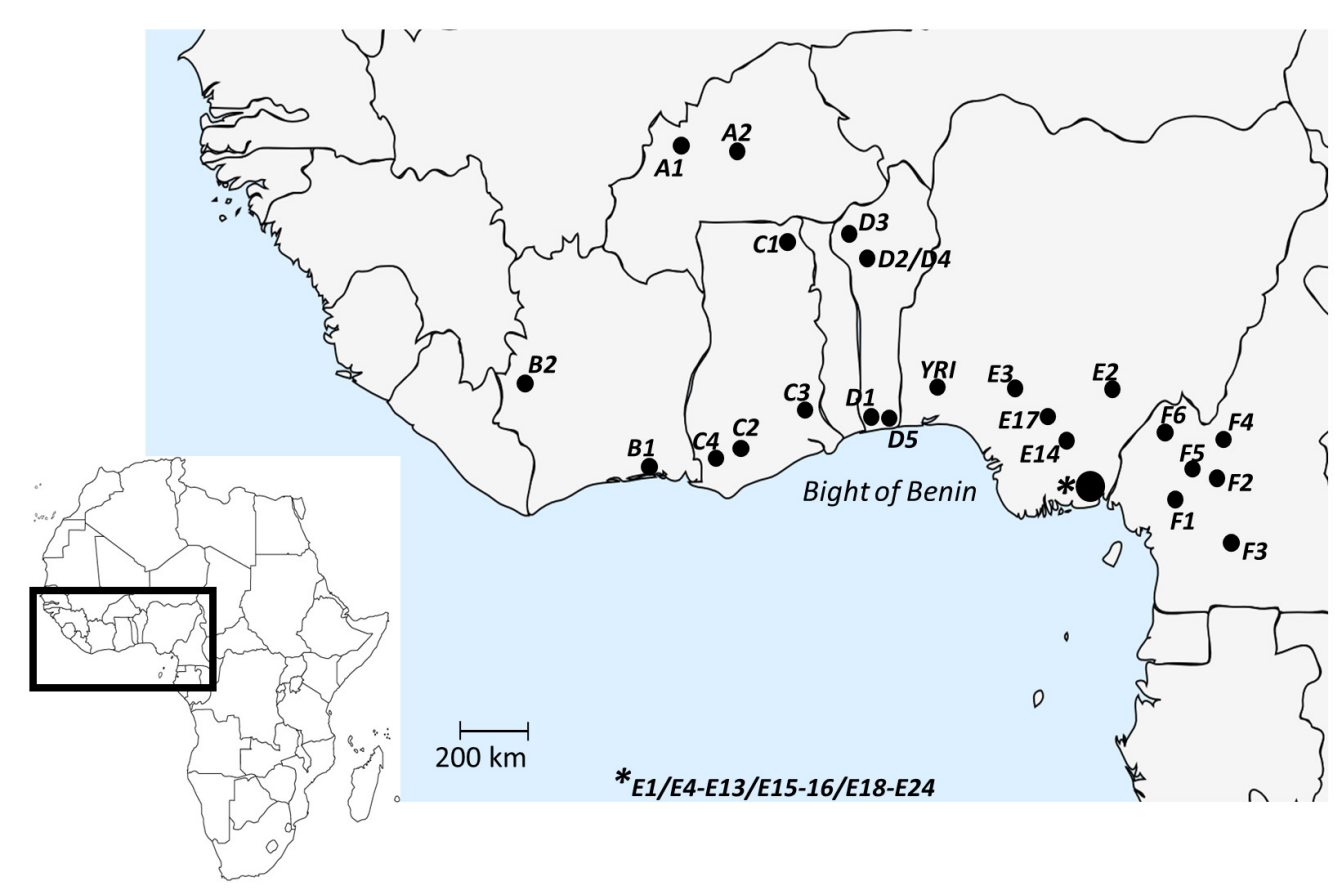
Geographic location of all population samples along the Bight of Benin and the rest of the West-African coast analysed in the study. The nomenclature and the references of the population samples are available in S1 Table.

One African region that is still poorly characterized genetically is the region along the Bight of Benin, defined as the area compassing East Ghana, Togo, Benin and Southwest Nigeria (Fig 1). The Bight of Benin is also known as the ‘Slave coast’ since its ports, including the well-known example of Ouidah [21], were actively used in the slave trade between the 16^th^ and 19^th^ centuries [22]. In total 2,340,000 individuals were transported from the Bight of Benin, *circa* 22% of all the African slaves sent to the Americas [23]. Notwithstanding the high diversity of ethnicities, languages (Niger Congo as well as Nilo-Saharan [1]) and subsistence modes [24] that characterize the Bight of Benin, many population genetic studies have used only one sample from this region, the HapMap sample YRI from the Yoruban population in South-western Nigeria [25]. The representativeness of YRI in population genetic studies has been assessed using autosomal loci and Y-STRs, however, only one–also Yoruban–West-African population was used for this purpose, so the representativeness of YRI for the whole of West Africa remains in question [6]. Examples of Y-chromosomal studies where such representativeness issues may be important are the recent phylogeographic refinement and large-scale genotyping of the Y-chromosomal haplogroup E (E-M96) in Africa [15], the world-wide forensic study of the new standard Y23 Y-STR kit [26] and a study of the origins of African Americans using uniparental markers [27]. Only one Y-chromosomal study with several populations from the region along the whole Bight of Benin has been realised so far [28], but this was based on a limited number of Y-chromosomal markers, namely seven Y-SNPs and four Y-STRs, before the publication of the Y-Chromosome Consortium (YCC) phylogeny [29]. Therefore, new samples and a state-of-the-art meta-analysis of already published samples would be a useful contribution to characterizing the Y-chromosomal landscape in the Bight of Benin region.

In this study, we performed an up-to-date Y-chromosomal genotyping of four Beninese populations which were sampled more than two decades ago [24, 30, 31]. We compared the Y-SNP and Y-STR data of these samples with other Y-chromosomal data from the Bight of Benin and surrounding areas published in the literature (till July 2015). As each published study used its own Y-SNP panels based on different version of the Y-chromosomal phylogeny, we developed an extensive method to combine new and old Y-SNP data without losing information. Finally, we also analysed the Y-STR and Y-SNP data of the YRI HapMap sample to test its regional representativeness [6, 25]. The Y-chromosomal haplogroup assignment of the YRI individuals was done by analysing whole genome SNP calling data from the 1000 Genomes project and the open access Complete Genomics database. As the assignment was done differently from Y-SNP typing, it was analysed separately from the other populations from the Bight of Benin region.

## Materials & Methods

### Y-chromosomal genotyping

In this study, the Y-chromosome was genotyped in 120 unrelated DNA-donors from four Beninese populations which were sampled for earlier genetic studies [24, 31], namely the Bariba (N = 34; population sample D2), Berba (N = 13; D3), Dendi (N = 37; D4) and Fon (N = 36; D5) (Fig 1; S1 Table). Appropriate written informed consent to anonymously use their data was obtained from all individuals during a collaborative project between Italy and Benin (with the Direction de l’Alimentation et de la Nutrition Appliquée, Porto Novo, as the Beninese partner). The ethics approval for the re-genotyping of these samples was provided by the ethical committee of the University of Rome Tor Vergata (Comitato Etico Indipendente; decision on 14 June 2011 for the “Protocollo di Studio Benin”, promoter: Olga Rickards). The DNA, which had been kept in Rome since 1989, was transferred to Leuven for an in-depth Y-chromosomal analysis. First, the samples were genotyped by using two multiplex SNaPshot mini-sequencing assays (Thermo Fisher Scientific) that allow for the hierarchical detection of 28 Y-SNPs defining the major worldwide Y-chromosomal haplogroups [32]. Then, the samples were assigned to specific Y-SNP assays to confirm the main haplogroup and to assign the subhaplogroup according to the Y-chromosomal tree of Karafet *et al*. [33], including recent updates [20, 34, 35] (see S1 Fig for the final phylogeny). A total of six multiplex systems with 40 Y-SNPs were developed using SNaPshot mini-sequencing assays according to previously published protocols [32, 36]. The protocols to perform these Y-SNP multiplex systems are explained in Larmuseau *et al*. [37] or available from authors upon request. Finally, a set of 42 Y-STRs, including 23 loci of the PowerPlex^®^ Y23 System (Promega Corporation) [26] and 19 widely used Y-STR loci within in-house developed multiplexes [37, 38], were genotyped for each DNA donor. The results of the PowerPlex^®^ Y23 System of 52 DNA-donors from population sample D2 and D5 were already included in a meta-analysis by Purps *et al*. [26]. The Y- chromosomal data for all 120 donors have been submitted to the open-access Y-STR Haplotype Reference Database (YHRD, www.yhrd.org): accession number YA003889.

### Statistical analysis

For the population genetic analysis based on Y-chromosomal haplogroup frequencies, published Y-SNP data for 39 population samples from the Western coast of Africa were collected (see geographical position of the samples in Fig 1; see references and characteristics of the samples in S1 Table). We selected only those southern Nigerian population samples with a minimal size of 35 unrelated DNA-donors due to the large amount of available data from this relatively small area. Different Y-chromosomal phylogenies were used within the various published studies on West-African populations, e.g. the phylogeny used by Ansari-Pour *et al*. [39] was based on a limited SNP-panel in comparison with the phylogeny used by de Filippo *et al*. [18]. Therefore, five different databases, each based on a particular phylogeny (S1–S5 Figs), were established to allow comparison between the four Beninese population samples with as many other populations as possible, and without losing information about SNPs that define Y-chromosomal lineages. In some instances, even when not all Y-SNPs featured in a particular phylogeny were genotyped in a study, the data of that study could be analysed based on that particular phylogeny; this was possible (a) when phylogenetically equivalent Y-SNPs were genotyped based on global Y-chromosomal trees [40–42], or (b) when no samples of that study were assigned to more basal (sub)haplogroups than the subhaplogroups described by the non-genotyped Y-SNPs. The subhaplogroups were always called using the nomenclature proposed by van Oven *et al*. [40]. Additionally, one database was prepared based on the phylogeny that made it possible to include all the collected populations (S7 Table; S6 Fig). Subhaplogroup frequencies were estimated and compared between populations for all databases. Pairwise *F*_*ST*_ values between the populations were estimated using arlequin v.3.1 [43]. Significance of population subdivision based on Y-SNP data was tested using a permutation test implemented in R [44], as developed in Larmuseau *et al*. [45], and the Bonferroni correction for multiple testing was applied to all *p*-values [46]. Principal component analyses (PCAs) were performed with the vegan package in R as a clustering analysis of the populations, including a *biplot* that plots on the same plane the vectors representing the contribution of each of the original variables to these components. The correspondence analyses (CA) based on the Y-chromosome haplogroup frequencies were performed using the CA package in R.

The Y-STR haplotypes of the four Beninese population samples were compared with each other. First, median-joining haplotype networks for each subhaplogroup within the main E-haplogroup (E-M96) were constructed based on all 30 genotyped single-copy Y-STRs (multi-copy Y-STRs are not suitable for network construction) with network version 4.5.1.0 [47] using the weighting scheme described by Qamar *et al*. [48] and the locus-specific mutation rates reported by Ballantyne *et al*. [49]. Second, population differentiation and pairwise *F*_*ST*_ and *R*_*ST*_ values based on all 30 single-copy Y-STRs were calculated between the four Beninese population samples; pairwise *F*_*ST*_ and *R*_*ST*_ values were also calculated from 15 single Y-STRs in common between eight West-African population samples from Ivory Coast (B1-2), Ghana (C1) and Benin (D1-5) and from 14 single Y-STRs in common between 14 West-African population samples from Ivory Coast (B1-B2), Ghana (C1), Benin (D1-D5), Nigeria (E1-E3) and Cameroon (F1-F3). The Y-STR allele nomenclature of the YHRD database [50] was used to compare the Y-STR data from the different studies. All values were estimated using arlequin v.3.1 [43] and tested for statistical significance by means of random permutation of samples in 10,000 replicates. The sequential Bonferroni correction was applied to correct significance levels for multiple testing [46]. The pairwise *F*_*ST*_ and *R*_*ST*_ values were used to visualize population structure with a two-dimensional classical multidimensional scaling (CMDS) plot with the function *cmdscale* in the vegan package for R.

### Y-chromosomal analysis of whole-genome sequences in the YRI sample

Whole-genome SNP calling data of 48 Yoruba males from Ibadan (Nigeria; HapMap sample YRI) were collected from the 1000 Genomes project–the pilot phase [51] and phase 1 [52]–and the public database of Complete Genomics [53]. Y-chromosomal haplogroups were assigned using AMY-tree software [42, 54] using the latest published phylogenetic tree for this tool (including the most recent updates) [41, 55]. When the Matthews correlation coefficient (MCC) in the AMY-tree analysis was lower than 0.50, the quality of the haplogroup assignment was assumed to be too low and the individual was removed from further analysis. When the same individual was sequenced in different projects, the whole-genome sequence with the highest MCC was selected for further analysis. The frequencies of the Y-chromosomal variants were compared between the YRI sample and the other West-African populations by estimating pairwise *F*_*ST*_ values and performing PCA and CA as described earlier. Finally, publicly available Y-STR data of 30 YRI individuals were also collected [6]. Pairwise *F*_*ST*_ values based on the 15 and 14 single Y-STRs shared between YRI and, respectively, the eight and 14 West-African population samples, were calculated as mentioned before. The pairwise *F*_*ST*_ values were also used to visualize population structure with CMDS plots as described above.

## Results

### Y-chromosomal haplogroup frequencies and population differentiation

Among the 120 DNA-donors composing the four Beninese population samples genotyped (S2 Table) according to the phylogeny given in S1 Fig, twelve Y-chromosomal subhaplogroups were observed. Most of the individuals belonged to one of four subhaplogroups: E-M191*, E-U174*, E-U209* and E-U290*. Except for the Berba sample (D3), the most frequent haplogroup was E-M174* (total frequency for the four Benin samples = 31.7%). Pairwise F_*ST*_-values were never significant between the populations, except for the values between Berba (D3) and Bariba (D2) and between Berba (D3) and Fon (D5) (S8A Table). The CA-plot and the PCA-plot showed that D3 was the most differentiated population sample whereas Dendi (D4) and Fon (D5) were the samples most similar to each other (S7 Fig).

Based on the five additional phylogenies defined (S2–S5 Figs), the Y-SNP data of the Beninese samples were compared with published data for other West African populations. First, Y-SNP data of 573 individuals from 11 population samples of Ghana, Benin, Nigeria and Cameroon were compared according to the phylogeny presented in S2 Fig (see S3 Table for haplogroup frequencies). The most aberrant pattern was found in the Ghanaian population (C1) with no samples belonging to E-U290, despite this haplogroup’s high frequency in the other populations (S3 Table). The outlier position of C1 was observed in the *F*_*ST*_-values (S8A Table), the CA and the PCA (S7 Fig).

**Fig 2:**
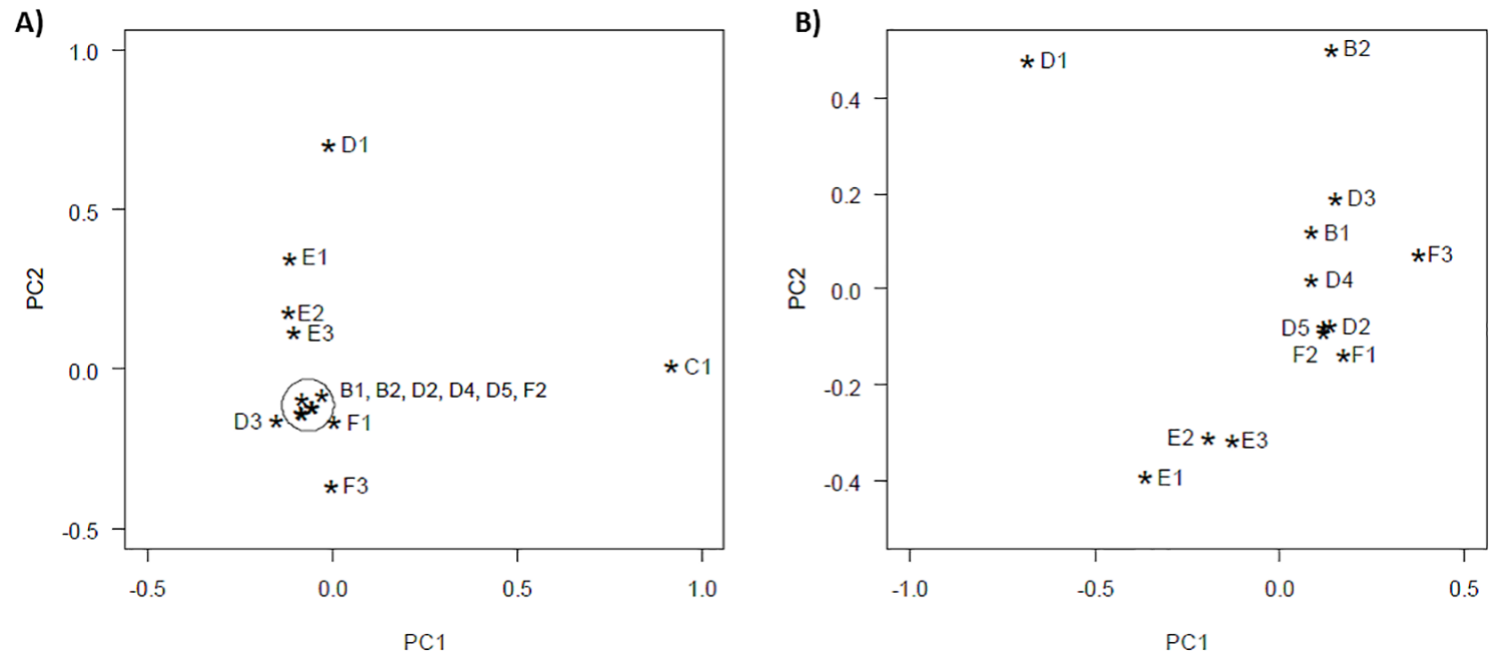
Principal component analysis (PCA), together with a biplot, of 14 West-African population samples, (a) inclusively and (b) exclusively the Ghanaian C1 population sample, based on the Y-SNP frequencies using the phylogeny given in S3 Fig. The cumulative proportion of plot (a) is 0.92 for the first two principal components (PC1: 0.83; PC2: 0.09), and of plot (b) is 0.81 for the first two principal components (PC1: 0.45; PC2: 0.36). The nomenclature and the references of the population samples are available in S1 Table.

Second, Y-SNP data of 741 individuals from 14 population samples within Ivory Coast, Ghana, Benin, Nigeria and Cameroon were compared according to the phylogeny presented in S3 Fig (see S4 Table for haplogroup frequencies). The population sample from Ghana (C1) and the Benin population sample from Brucato *et al*. [27] (D1) were the most differentiated based on the subhaplogroup frequencies (S4 Table), the *F*_*ST*_-values (S8A Table) and the PCA plot (Fig 2). In the CA-plot (S7 Fig) population samples D3 and B2 were also differentiated, next to samples C1 and D1. The Nigerian populations (E1-E3) always clustered together, as did the Cameroonian populations (F1-F3).

Third, Y-SNP data of 991 individuals from 16 population samples from Burkina Faso, Ivory Coast, Ghana, Benin, Nigeria and Cameroon were compared following the phylogeny presented in S4 Fig (see S5 Table for haplogroup frequencies). Based on the *F*_*ST*_-values (S8C Table) and the PCA (S7 Fig), the population sample from Ghana (C1) and the additional Beninese population sample (D1) were the most differentiated in the analysis. No strongly outlying samples were observed in the CA-plot (S7 Fig).

Fourth, Y-SNP data of 2198 individuals from 38 populations of Ghana, Benin, Nigeria and Cameroon were compared using the phylogeny presented in S5 Fig (see S6 Table for haplogroup frequencies). The most differentiated populations based on the *F*_*ST*_-values (S8D Table), the CA- and PCA-plot (S7 Fig) were one Ghanaian (C1) and four Cameroonian population samples (samples F1 and F3 in the CA-plot and samples F5 and F6 in the PCA plot).

Finally, the Y-SNP data for 2616 individuals from all 43 West-African populations in the collection were compared following the phylogeny of S6 Fig. Nine subhaplogroups were observed with E-M96*, E-M2*, E-M191 and E-U175 as the most frequent examples (S7 Table). Based on the pairwise F_*ST*_-values (S8E Table), the CA- and PCA-plot with and without C1 (S7 Fig), the most differentiated population samples present in this database were those from Burkina Faso (A1-A2), the D1-sample from southern Benin in Abomey-Calavi, next to the Ghanaian C1-sample.

### Y-STR haplotype comparison and population differentiation

Due to DNA degradation a complete 42 Y-STR haplotype was not retrieved for some donors even after several attempts (S2 Table). The median-joining haplotype network of all Beninese Y-chromosomes belonging to haplogroup E (E-M96) showed some clear grouping of haplotypes which belonged to the same subhaplogroup, but also clustering which was not attributable to genotyped Y-SNPs (S8 Fig). Clusters of haplotypes belonging to subhaplogroups E-M2*, E-U174* E-M191*, E-M209* and E-U290* were found to be scattered around the network instead of clustering together (S8 Fig). To exclude SNP typing error as an explanation, the original SNP calls were verified by re-genotyping the samples having the most divergent haplotypes in this network analysis.

No pairwise *F*_*ST*_ values between the four Beninese population samples based on the 30 single-copy Y-STRs were significant (S9A Table). In contrast, most of the pairwise *F*_*ST*_ values based on 15 Y-STRs in common between eight West-African population samples (S9B Table), and based on 14 Y-STRs in common between 14 West-African population samples (S9C Table), were significant. The CMDS plot visualizing population structure based on the pairwise *F*_*ST*_ values showed no pattern between the four Beninese population samples using 30 Y-STRs (not shown). Using the 15 Y-STRs, the CMDS plot showed that most of the eight analysed West-African population samples clustered together, except three samples which were strongly differentiated from the other samples and from each other, namely B1, C1 and D3 (S9A Fig). Using the 14 Y-STRs most of the 14 analysed West-African population samples also clustered closely together in the CMDS, except for the three population samples C1, D3 and F1 (S9B Fig). All the stress values of the CMDS were smaller than 0.10, indicating a good fit. No differences were found between the calculated *F*_*ST*_ and *R*_*ST*_ values (*R*_*ST*_ results are not shown).

### Data from whole-genome sequences in Yoruba

**Fig 3:**
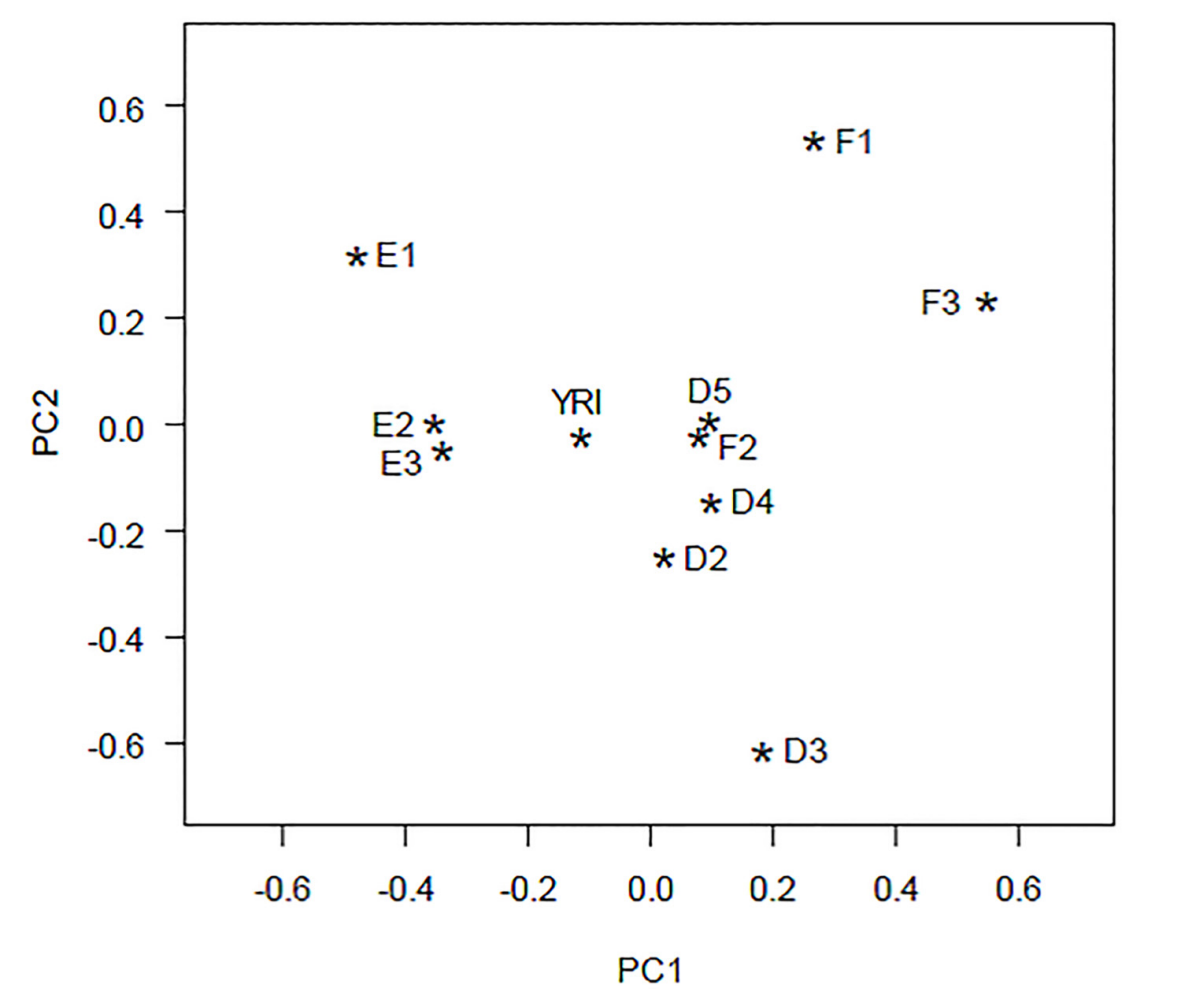
Principal component analysis (PCA) of ten West-African population samples and the HapMap YRI sample based on the Y-SNP frequencies using the phylogeny given in S2 Fig. The cumulative proportion of the plot is 0.90 for the first two principal components (PC1: 0.63; PC2: 0.27). In this analysis the Ghanaian C1 population sample was excluded from the analysis. The nomenclature and the references of the population samples are available in S1 Table.

The Y-chromosomal haplogroup was assigned in 48 Yoruba whole-genome sequences (WGS) using the most recent update of the Y-chromosomal phylogeny for the AMY-tree software (S10 Table). The MCC value of the AMY-tree analysis of one individual was <0.50, hence this individual was removed from further analysis. Despite the most recent update of the Y-chromosomal phylogeny, which contains a larger number of Y-SNPs within haplogroup E (E-M96) in comparison with the phylogeny given in S1 Fig, the particular haplogroups determined for the Yoruba sample were phylogenetically almost synonymous to those found in the four Beninese samples. As the MCC values were always < 0.95, except for the three samples which were sequenced by Complete Genomics, caution has to be taken about the determined haplogroups as the quality of the full sequence analysis was not optimal and relevant Y-SNP calls for the determination of the haplogroup could have been missed [42]. Nevertheless, the frequencies of the subhaplogroups in the WGS sample are highly comparable with the ones in Benin and Nigeria, based on the *F*_*ST*_-values (S11A and S11B Table), the CA (S10 Fig) and PCA (Fig 3, S10 Fig) analyses. Finally, *F*_*ST*_-values between YRI and the other West-African samples were calculated based on 15 and 14 Y-STRs (S11C and S11D Table) and used to visualize in CMDS plots (S11 Fig). Within these CMDS plots YRI clustered together with the other samples from the Bight of Benin and neighbouring regions, excluding the already mentioned outlier population samples B1, C1, D3 and F1. All the stress values of the CMDS-plots were smaller than 0.10, indicating a good fit.

## Discussion

This study reports for the first time the Y-chromosomal landscape in the West-African region along the whole Bight of Benin by analysing several population samples. Novel state-of-the-art Y-chromosomal haplotypes as well as several already published data (till July 2015) were analysed for this purpose. An extended method to compare both novel and already published Y-chromosomal data was used as each particular published study adopted its own specific Y-SNP panel based on a certain phylogeny and/or differed in the number of genotyped Y-STRs. In most meta-analyses, researchers select one specific phylogeny with a trade-off between the number of included Y-SNPs and the amount of already published population samples. To avoid this trade-off, we defined five phylogenies and one minimum phylogeny, so that we had six datasets with different numbers of Y-SNP and population samples included. Based on our study, analysing these different datasets was clearly the best strategy instead of selecting only one particular phylogeny, as the choice of Y-SNPs might have influenced the interpretation of the results. To summarize all these different analyses: the fewer Y-SNP-defined haplogroups that were taken into account, the less informative was the geographical structuring between the population samples, and the less marked the outlier position of some samples. Finally, we also included haplogroup frequency data from the whole-genome SNP data of the HapMap sample YRI (South-western Nigeria) in the analysis. Since SNP calling of the whole-genome sequence data for YRI was not optimal due to a low sequence coverage in the 1000 Genome project [52], the assignment of most of the YRI samples to a Y-chromosomal subhaplogroup was also suboptimal [42, 54]. Therefore, the YRI data were analysed separately from the SNP-typing data.

### Y-chromosomal diversity in West-African Bight of Benin

As expected based on pan-African population genetic studies, almost all individuals (95%) sampled in the region along the Bight of Benin belonged to Y-chromosomal haplogroup E (E-M96) [56]. Only a limited number of samples was assigned to other haplogroups, namely Y(xBCDEF-M91), B (B-M60) and R (R-M207) (S3–S7 Tables). Some individuals from the Benin, Ghana and Nigeria were assigned to Y(xBCDEF-M91), together with some individuals from Burkina Faso and Cameroon. The single individual of this haplogroup that was SNP-typed in more detail belonged to subhaplogroup A3-M13 and was observed in the southern Beninese sample of the Fon population (D5). Haplogroup B (B-M60) was only observed in Burkina Faso, Nigeria and Cameroon. Although the distribution map of haplogroup B (B-M60) in Scozzari *et al*. [57] also covers Ghana and Benin, the haplogroup was not detected in the analysed population samples within those countries. Due to the low frequencies, there is still a limited knowledge of the diversity and distribution of the root haplogroups in West-Africa [57, 58]. This is also illustrated by the recent discovery of the lineage A00 in a Bantu-population from North Cameroon, which added an extremely ancient root to the Y-chromosomal phylogenetic tree [20]. Finally, two individuals from one Beninese sample of the Bariba population (D2) were assigned to haplogroup R-V88 based on the phylogeny of S1 Fig. Using the other phylogenies it was not possible to assign samples to a phylogenetic level deeper than haplogroup R (R-M207) or P (P-P295*). This was the case for individuals sampled in Southern Ghana, Nigeria and Cameroon (frequency <5%), but which likely could have been assigned to R-V88 with more Y-SNP typing efforts. According to Cruciani *et al*. 2010 [35], R-V88 is the result of a mid-Holocene trans-Saharan connection and is linked to Chadic languages, as the haplogroup has a frequency of 95% in a range spanning Central Sahel up to Northern Cameroon, Northern Nigeria, Chad and Niger, but it drops drastically to 0–5% immediately south of this region. The low frequency of R-V88 in the Bight of Benin fits completely with this earlier observation.

Most of the West-African samples assigned to haplogroup E (E-M96) were phylogenetically further defined within four main subhaplogroups, namely E-M191*, E-U174*, E-U209* and E-U290* based on the most extended phylogeny given in S1 Fig (S3 Table). These haplogroups have a pan-African distribution which makes it difficult to pinpoint a clear geographical origin of a certain individual carrying such a haplogroup [59]. Nevertheless, the current list of known Y-SNPs in publications on African populations are still subject to ascertainment bias, since only a limited number of African Y-chromosomes have been fully sequenced [42]. Therefore, the current phylogenies are most likely not yet explanatory of the phylogeographic variation present in West-African populations [60]. This is visible in the haplotype networks based on 30 single copy Y-STR loci of the four Beninese samples assigned to haplogroup E (E-M96) and to one of its main subhaplogroups (S8 Fig), where clear sub-structuring was observed, although not yet supported by specific Y-SNPs. Even though the phylogeny of S1 Fig was not the most comprehensive one available, the haplogroup assignment of the YRI sample from SNP calling data was realized by the AMY-tree software using the most up-to-date phylogenies [41], and the samples were assigned to the same haplogroups as defined by the phylogeny given in S1 Fig. Therefore, even the latest phylogenies described in the literature do not yet provide coverage of all sub-structuring patterns in the Y-STR haplotype networks. To cover all relevant lineages in (West-)Africa, studies like Hallast *et al*. [60] which investigated (almost) full Y-chromosomes of Europeans, are required. A recent study by Trombetta *et al*. [15] did attempt this; however, the (almost) fully sequenced Y-chromosomes assigned to haplogroup E (E-M96) were not sufficiently dispersed geographically to provide a global view on the African diversity within this haplogroup. For the Bight of Benin, only Y-chromosomes of the HapMap YRI sample were included in the analysis. These studies can be crucial, as even a small increase in the number of phylogenetically relevant Y-SNPs can reveal novel patterns of genetic variation in Europe and even more so in Africa [61, 62]. Nevertheless, based on the currently used phylogenies, it was already possible to study differentiation patterns between the population samples along the Bight of Benin and the neighbouring regions.

### Population differentiation along the Bight of Benin

With the exclusion of some samples which were strong outliers in the population genetic analysis, the population differentiation between the samples along the Bight of Benin is mainly determined by geography, notwithstanding the many ethnic-linguistic groups and subsistence lifestyles in this region. The Mantel-tests performed to test the correlation between geographical and genetic distances were significantly positive for the population samples along the Bight as well as for all samples in the analysis (p>0.01; results not shown) [63]. No specific clustering or strong differentiation was found between populations based on language differences, including the sample of the Dendi population (sample D4) which speaks a Nilo-Saharan language (unique for the region) and the Bantu-speaking population samples, although some studies claimed a specific haplogroup association with these Bantu-populations [18]. Although language and geography are linked to each other, we can state that the geographic proximity seems to be the best predictor of genetic affinity. The population genetic pattern in Western Africa is in any case a palimpsest, and the result of a complex historical background and socio-cultural characteristics. To further unravel it, specific sampling designs have to be realised since the current samples along the Bight of Benin are certainly not sufficient. Nonetheless, this study provides a clear synopsis of the Y-chromosomal variation and differentiation which is expected within this region, and this will be useful in future population genetic and forensic studies.

Although most population samples showed homogenization across the Bight of Benin, we detected some clear outlier samples in the analysis. In a meta-analysis samples may differentiate from each other due to a different ascertainment of the DNA donors, and thus may occupy an outlier position within the population genetic analysis. For the region along the Bight of Benin, two population samples in the analyses were clearly outliers, namely one sample from northern Ghana (C1) and another from southern Benin in Abomey-Calavi (D1), analysed by Sanchez-Faddeev *et al*. [4] and Brucato *et al*. [27], respectively.

The outlier position of the sample from northern Ghana (C1) in the analysis based on Y-STRs as well as on Y-SNPs was most likely due to the specific sampling design of the study of Sanchez-Faddeev *et al*. [4], aimed at testing the pattern of variation between clans within one single community. Only three different subhaplogroups were observed (S3 Table) and many DNA donors were related to each other as sometimes even two members of one household were sampled. As they detected a strong differentiation between the clans, the authors claimed that social factors on micro-geographical scale may interfere with processes on regional and even continental scales, thus biasing the interpretation of macro-geographical genetic differentiation in Western Africa [4]. The large difference of Y-chromosomal variation in one community in comparison with the regional variation in Western Africa is in sharp contrast with the situation observed in other well-studied areas, e.g. small communities of Western Europe exhibited the same Y-chromosomal variation as a similar sample of individuals from the whole region (defined by a circle with diameter 30 km) in the late Middle Ages [64].

The second sample which was highly differentiated from the other Bight of Benin samples in the meta-analysis, was the one of the Fon population–including some individuals of the Yoruba, Goun and Tofin populations–from Abomey-Calavi in Southern Benin (D1). There was a clear difference in subhaplogroup frequencies (S3 Table), but surprisingly, no significant difference was observed based on the Y-STR loci (S9 Fig). As Abomey-Calavi is one of the main cities in the south of Benin and the DNA donors were unrelated to each other, no local influences were expected. We also genotyped another sample of the Fon population (D5) which was taken from another main city Cotonou, less than 12 km away from Abomey-Calavi, and based on history or social differences, no genetic differentiation was expected between the two samples [65]. Therefore, a clear reason for the outlying position of sample D1 in the analysis is unknown as it can be due to sampling or technical issues or to local differentiation due to isolation. Since sample D1 was clearly an outlier in comparison with the other samples of the Bight of Benin based on Y-chromosomal haplogroup frequencies, our approach shows that the use of only this sample to represent the Bight as was done in the study of Brucato *et al*. [27] to study the origins of modern African Americans based on uniparental markers, could have biased the analysis.

These two examples of outlier samples stress that when a particular population sample is used as a reference for a certain African region within a macro-geographical population genetic analysis, this sample has to be also representative for that particular region and not subjected to a local, isolated situation or flawed sampling design. Therefore, the result that the commonly used HapMap sample YRI of the Yoruba population from Ibadan in West-Nigeria was highly representative for Western Africa based on the Y-chromosomal haplogroups and Y-STRs, was a crucial one. Within the PCA and CA-plots, the YRI sample was clearly positioned between the Beninese and the Nigerian population samples (S10 and S11 Figs). This shows that a previous claim that the YRI sample was representative for Western Africa using Y-STRs and autosomal data is here confirmed, although this was done in the previous attempt by comparing the YRI data with only one other–also Yoruba–West-African population sample [6]. As the representativeness was tested here with more samples from Western Africa, it confirms the claim that the YRI can be used for standard population genetic and forensic studies, although the ascertainment of the HapMap samples was done for medical reasons [25]. Therefore, the uniquely powerful geographical information carried by the Y-chromosome makes it, together with the mitochondrial genome, an important locus to test the representativeness of a certain sample even in the genomic era, especially in poorly investigated areas like Africa which displays a complex picture with a multitude of languages, ethnic groups, intra-communal sub-structuring and lifestyles.

## Supporting Information

S1 Fig Schematic representation of the phylogeny of Y-chromosomal subhaplogroups based on the binary markers which were analysed for the four Beninese population samples. The nomenclature of the subhaplogroups is based on the terminal mutation that defines them, as proposed in van Oven *et al. [40]*. *Paragroups: Y-chromosomes not defined by any downstream-examined mutation.
(TIF)

S2 Fig Schematic representation of the phylogeny of Y-chromosomal subhaplogroups based on the binary markers which could be analysed for eleven West-African population samples given in S1 Table. The nomenclature of the subhaplogroups is based on the terminal mutation that defines them, as proposed in van Oven *et al. [40]*. *Paragroups: Y-chromosomes not defined by any downstream-examined mutation.
(TIF)

S3 Fig Schematic representation of the phylogeny of Y-chromosomal subhaplogroups based on the binary markers which could be analysed for 14 West-African population samples given in S1 Table. The nomenclature of the subhaplogroups is based on the terminal mutation that defines them, as proposed in van Oven *et al. [40]*. *Paragroups: Y-chromosomes not defined by any downstream-examined mutation.
(TIF)

S4 Fig Schematic representation of the phylogeny of Y-chromosomal subhaplogroups based on the binary markers which could be analysed for 16 West-African population samples given in S1 Table. The nomenclature of the subhaplogroups is based on the terminal mutation that defines them, as proposed in van Oven *et al. [40]*. *Paragroups: Y-chromosomes not defined by any downstream-examined mutation.
(TIF)

S5 Fig Schematic representation of the phylogeny of Y-chromosomal subhaplogroups based on the binary markers which could be analysed for 38 West-African population samples given in S1 Table. The nomenclature of the subhaplogroups is based on the terminal mutation that defines them, as proposed in van Oven *et al. [40]*. *Paragroups: Y-chromosomes not defined by any downstream-examined mutation.
(TIF)

S6 Fig Schematic representation of the phylogeny of Y-chromosomal subhaplogroups based on the binary markers which could be analysed for 43 West-African population samples given in S1 Table. The nomenclature of the subhaplogroups is based on the terminal mutation that defines them, as proposed in van Oven *et al. [40]*. *Paragroups: Y-chromosomes not defined by any downstream-examined mutation.
(TIF)

S7 Fig Correspondence analyses (CA) and principal component analysis (PCA) within the meta-analysis of the West-African samples. The nomenclature and the references of the population samples are available in S1 Table. With a) CA and b) PCA, together with a *biplot*, of the four–in this study re-genotyped–Beninese population samples based on the Y-SNP frequencies using the phylogeny given in S1 Fig. The cumulative proportion is 0.96 for the first two principal components (PC1: 0.82; PC2:0.14). With c) CA and d-e) PCA of the eleven West-African population samples with (d) and without (e) sample C1, all based on the Y-SNP frequencies using the phylogeny given in S2 Fig. The cumulative proportion of plot (d) is 0.98 for the first two principal components (PC1: 0.92; PC2: 0.06), and of plot (e) is 0.92 for the first two principal components (PC1: 0.64; PC2: 0.28). With f) CA and g-h) PCA of the 14 West-African population with (g) and without (h) sample C1, all based on the Y-SNP frequencies using the phylogeny given in S3 Fig. The cumulative proportion of plot (g) is 0.92 for the first two principal components (PC1: 0.83; PC2: 0.09), and of plot (h) is 0.81 for the first two principal components (PC1: 0.45; PC2: 0.36). With i) CA and j-k) PCA of 16 West-African population samples with (j) and without (k) sample C1, all based on the Y-SNP frequencies using the phylogeny given in S4 Fig. The cumulative proportion of plot (j) is 0.87 for the first two principal components (PC1: 0.74; PC2: 0.13), and of plot (k) is 0.79 for the first two principal components (PC1: 0.54; PC2: 0.25). With l) CA and m-n) PCA of 38 West-African population samples with (m) and without (n) sample C1, all based on the Y-SNP frequencies using the phylogeny given in S5 Fig. The cumulative proportion of plot (m) is 0.86 for the first two principal components (PC1: 0.58; PC2: 0.30), and of plot (n) is 0.83 for the first two principal components (PC1: 0.70; PC2: 0.13). With o) CA and p-q) PCA of the 43 West-African population samples with (g) and without (h) sample C1, all based on the Y-SNP frequencies using the phylogeny given in S6 Fig. The cumulative proportion of plot (p) is 0.86 for the first two principal components (PC1: 0.58; PC2: 0.28), and of plot (q) is 0.81 for the first two principal components (PC1: 0.55; PC2: 0.26).
(PDF)

S8 Fig Median-joining haplotype network based on all 30 genotyped single-copy Y-STR haplotypes of individuals from the Beninese population samples which belong to haplogroup E (E-M96). The size of the circles are proportional to the haplotype frequency. The colour of the circles represents the subhaplogroup to which the haplotype belongs based on Y-SNP typing and based on the phylogeny given in S1 Fig.
(TIF)

S9 Fig Classical multidimensional scaling (CMDS) plot based on pairwise *F*_*ST*_ values a) between eight West-African population samples using 15 Y-STRs and b) between 14 West-African population samples using 14 Y-STRs. The nomenclature and the references of the population samples are available in S1 Table.
(TIF)

S10 Fig Correspondence analyses (CA) and principal component analysis (PCA) of the HapMap YRI sample and other West-African samples. The nomenclature and the references of the population samples are available in S1 Table. With a) CA and b) PCA, together with a *biplot*, of YRI and the four–in this study re-genotyped–Beninese population samples based on the Y-SNP frequencies using the phylogeny given in S1 Fig. The cumulative proportion is 0.95 for the first two principal components (PC1: 0.80; PC2:0.15). With c) CA and d-e) PCA of YRI and the eleven West-African population samples with (d) and without (e) sample C1, all based on the Y-SNP frequencies using the phylogeny given in S2 Fig. The cumulative proportion of plot (d) is 0.98 for the first two principal components (PC1: 0.92; PC2: 0.06) and of plot (e) is 0.90 for the first two principal components (PC1: 0.63; PC2: 0.27). With f) CA and g-h) PCA of YRI and 43 West-African population samples with (g) and without (h) sample C1, using the phylogeny given in S6 Fig. The cumulative proportion of plot (g) is 0.86 for the first two principal components (PC1: 0.58; PC2: 0.28), and of plot (h) is 0.81 for the first two principal components (PC1: 0.55; PC2: 0.26).
(PDF)

S11 Fig Classical multidimensional scaling (CMDS) plot based on pairwise *F*_*ST*_ values a) between eight West-African population samples and the HapMap YRI sample using 15 Y-STRs, and b) between 14 West-African population samples and the HapMap YRI sample using 14 Y-STRs. The nomenclature and the references of the population samples are available in S1 Table.
(TIF)

S1 Table Details of all 43 West-African population samples included in the Y-chromosomal meta-analysis. N, number of Y chromosomes; Phylogeny: data are analysed based on Y-chromosomal phylogeny/phylogenies as represented in S1–S6 Figs; Y-STRs: Y-STR data were available for analysis (+) or unavailable (-).
(XLSX)

S2 Table Y-chromosomal subhaplogroup and Y-STR data of the four–for this study re-genotyped–Beninese population samples D2-D5. The subhaplogroups were called using the phylogeny given in S1 Fig and the nomenclature proposed in van Oven *et al. [40]*. The nomenclature of the Y-STR alleles is conforming the one of the YHRD database (www.yhrd.org). ‘-’, no or a low-quality result was observed due to the severe DNA degradation of some samples.
(XLSX)

S3 Table Distribution (*N*) and frequency (*f*) of the Y chromosomal subhaplogroups within eleven West-African population samples which are analysed based on the phylogeny given in S2 Fig. The subhaplogroups were called using the nomenclature proposed in van Oven *et al. [40].* The highest subhaplogroup frequency is underlined for each population sample. The nomenclature and the references of the population samples are available in S1 Table.
(XLSX)

S4 Table Distribution (*N*) and frequency (*f*) of the Y chromosomal subhaplogroups within 14 West-African population samples which are analysed based on the phylogeny given in S3 Fig. The subhaplogroups were called using the nomenclature proposed in van Oven *et al. [40].* The highest subhaplogroup frequency is underlined for each population sample. The nomenclature and the references of the population samples are available in S1 Table.
(XLSX)

S5 Table Distribution (*N*) and frequency (*f*) of the Y chromosomal subhaplogroups within 16 West-African population samples which are analysed based on the phylogeny given in S4 Fig. The subhaplogroups were called using the nomenclature proposed in van Oven *et al. [40].* The highest subhaplogroup frequency is underlined for each population sample. The nomenclature and the references of the population samples are available in S1 Table.
(XLSX)

S6 Table Distribution (*N*) and frequency (*f*) of the Y chromosomal subhaplogroups within 38 West-African population samples which are analysed based on the phylogeny given in S5 Fig. The subhaplogroups were called using the nomenclature proposed in van Oven *et al. [40].* The highest subhaplogroup frequency is underlined for each population sample. The nomenclature and the references of the population samples are available in S1 Table.
(XLSX)

S7 Table Distribution (*N*) and frequency (*f*) of the Y chromosomal subhaplogroups within all 43 West-African population samples. The subhaplogroups were called using the phylogeny given in S6 Fig and the nomenclature proposed in van Oven *et al. [40].* The highest subhaplogroup frequency is underlined for each population sample. The nomenclature and the references of the population samples are available in S1 Table.
(XLSX)

S8 Table Pairwise *F*_*ST*_ values of (A) eleven West-African population samples based on the Y-chromosomal data using the phylogeny given in S2 Fig; (B) 14 West-African population samples based on the Y-chromosomal data using the phylogeny given in S3 Fig; (C) 16 West-African population samples based on the Y-chromosomal data using the phylogeny given in S4 Fig; (D) 38 West-African population samples based on the Y-chromosomal data using the phylogeny given in S5 Fig; (E) 43 West-African population samples based on the Y-chromosomal data using the phylogeny given in S6 Fig. *, p <0.05; **, p <0.01; ***, significant after Bonferroni correction. The shades of grey are different between *, ** and ***. The nomenclature and the references of the population samples are available in S1 Table.
(XLSX)

S9 Table Pairwise *F*_*ST*_ values of (A) the four Beninese population samples based on all 30 single-copy Y-STRs; (B) eight West-African population samples based on 15 common single Y-STRs; (C) 14 West-African population samples based on 14 common single Y-STRs. *, p <0.05; **, p <0.01; ***, significant after Bonferroni correction. The shades of grey are different between *, ** and ***. The nomenclature and the references of the population samples are available in S1 Table.
(XLSX)

S10 Table AMY-tree software results of the 48 Yoruban samples (YRI) from the Complete Genomics (CG) [53], 1000 Genomes pilot (1000G pilot) [51] and phase 1 (1000G phase 1) [52] projects. For each sample the determined subhaplogroup based on the latest published phylogeny for the AMY-tree software [41, 55], the Matthews correlation coefficient (MCC), the derived subhaplogroup based on the phylogeny given in S1 Fig (‘Subhaplogroup P1’), in S2 Fig (‘Subhaplogroup P2’) and in S6 Fig (‘Subhaplogroup P6’).
(XLSX)

S11 Table Pairwise *F*_*ST*_ values of (A) eleven West-African population sample and the HapMap YRI sample based on the Y-SNP data using the phylogeny given in S2 Fig; (B) 43 West-African population sample and the HapMap YRI sample based on the Y-SNP data using the phylogeny given in S6 Fig; (C) eight West-African population samples and the HapMap YRI sample based on 15 common single Y-STRs; (D) 14 West-African population samples and the HapMap YRI sample based on 14 common single Y-STRs. *, p <0.05; **, p <0.01; ***, significant after Bonferroni correction. The shades of grey are different between *, ** and ***. The nomenclature and the references of the population samples are available in S1 Table.
(XLSX)
