# Supplementary figures and images for: The Paternal Landscape along the Bight of Benin – Testing Regional Representativeness of West-African Population Samples Using Y-Chromosomal Markers
Source: PLoS One. 2015 Nov 6;10(11):e0141510. doi: 10.1371/journal.pone.0141510 (PMC4636292; doi:10.1371/journal.pone.0141510)

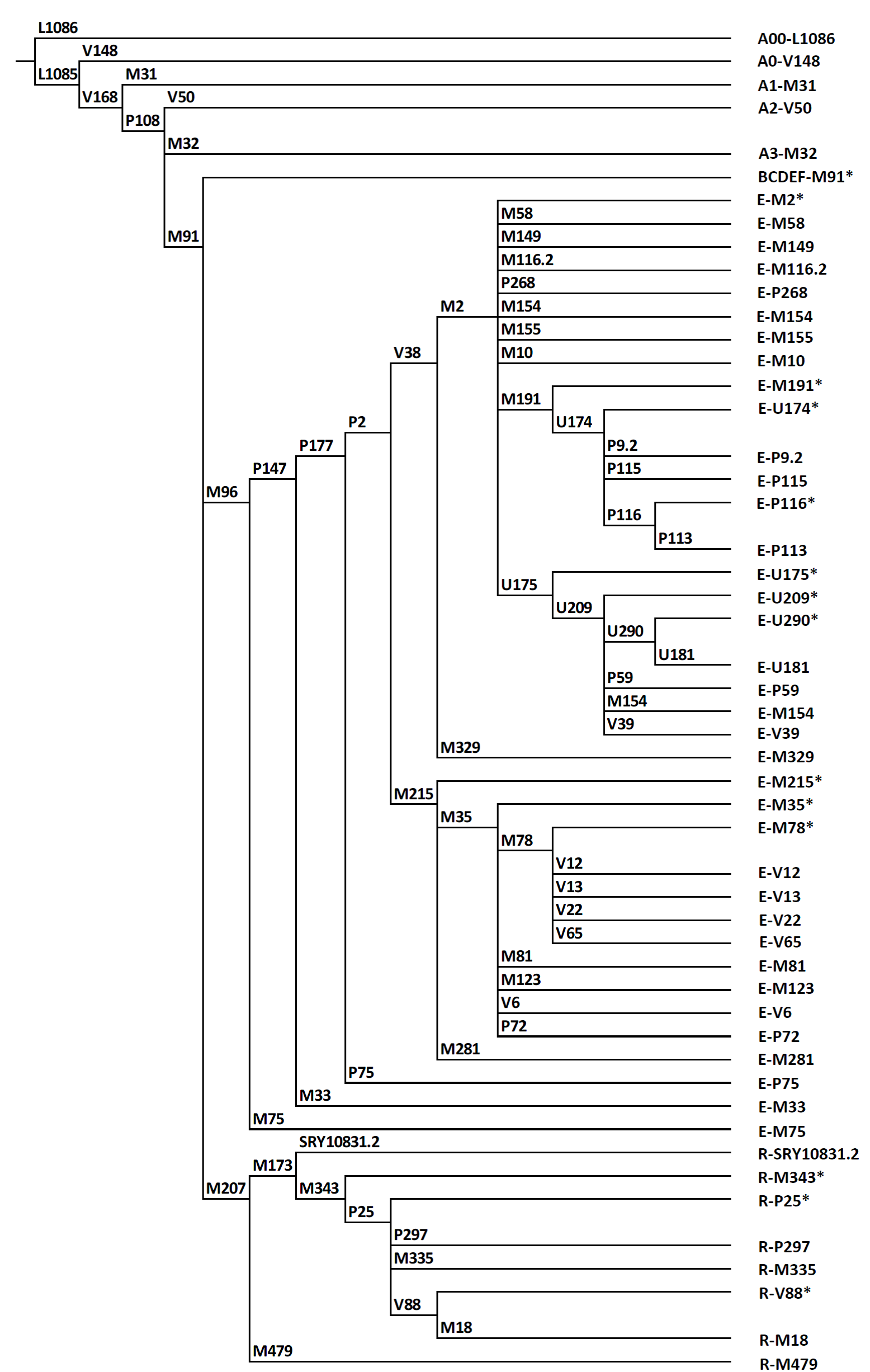

Supplement: S1 Fig — The nomenclature of the subhaplogroups is based on the terminal mutation that defines them, as proposed in van Oven et al. [40]. *Paragroups: Y-chromosomes not defined by any downstream-examined mutation. (TIF) [file pone.0141510.s001.tif]

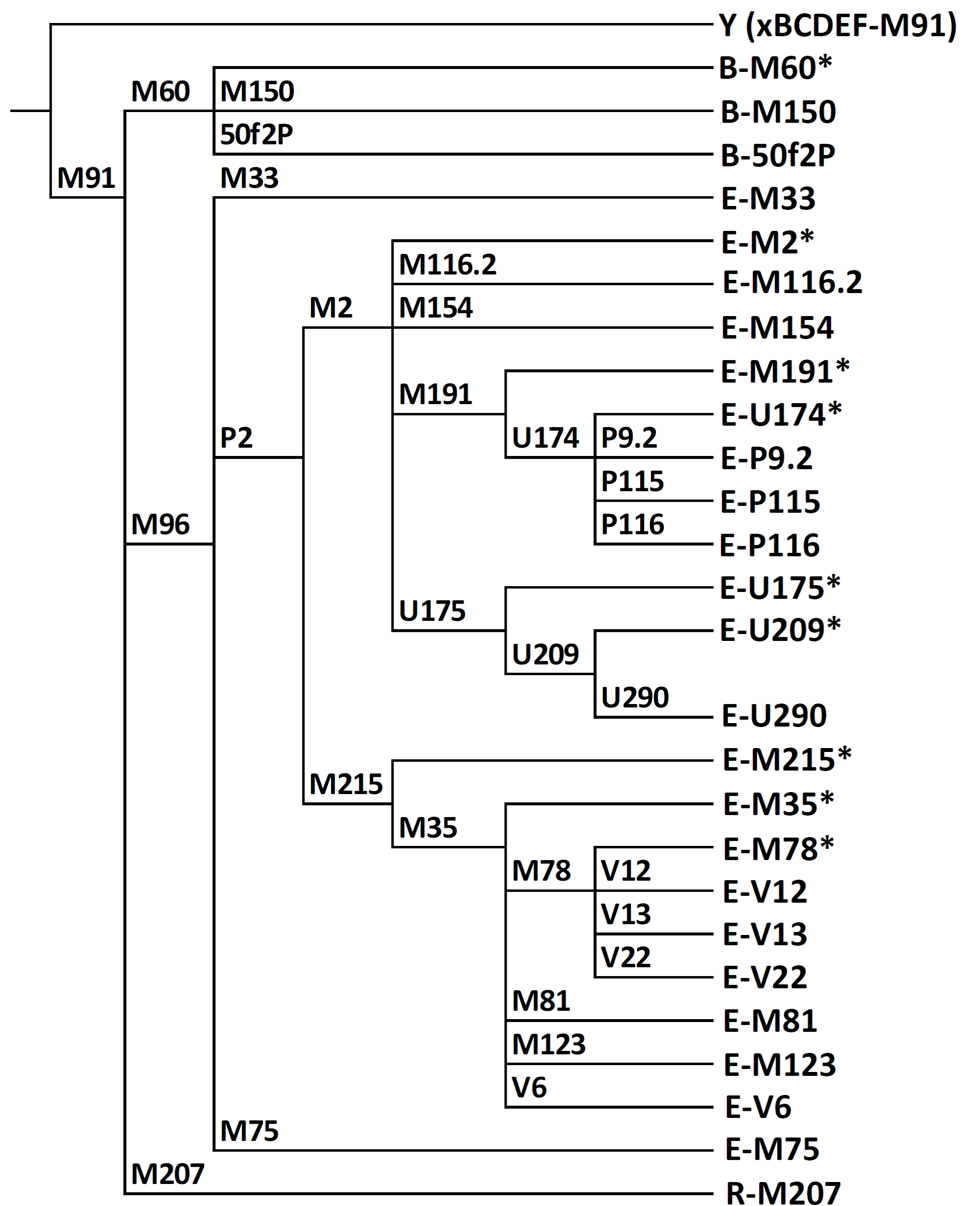

Supplement: S2 Fig — The nomenclature of the subhaplogroups is based on the terminal mutation that defines them, as proposed in van Oven et al. [40]. *Paragroups: Y-chromosomes not defined by any downstream-examined mutation. (TIF) [file pone.0141510.s002.tif]

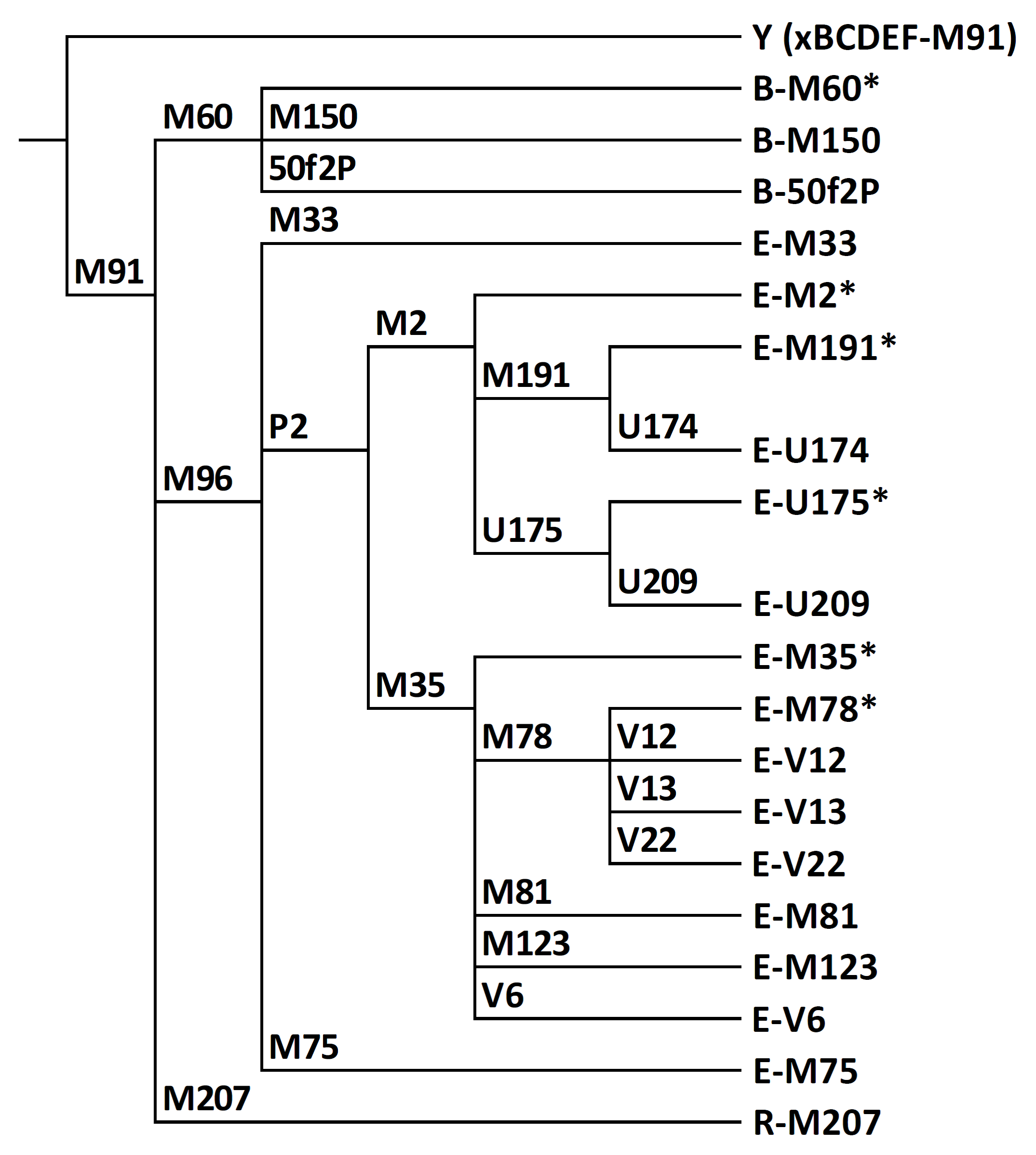

Supplement: S3 Fig — The nomenclature of the subhaplogroups is based on the terminal mutation that defines them, as proposed in van Oven et al. [40]. *Paragroups: Y-chromosomes not defined by any downstream-examined mutation. (TIF) [file pone.0141510.s003.tif]

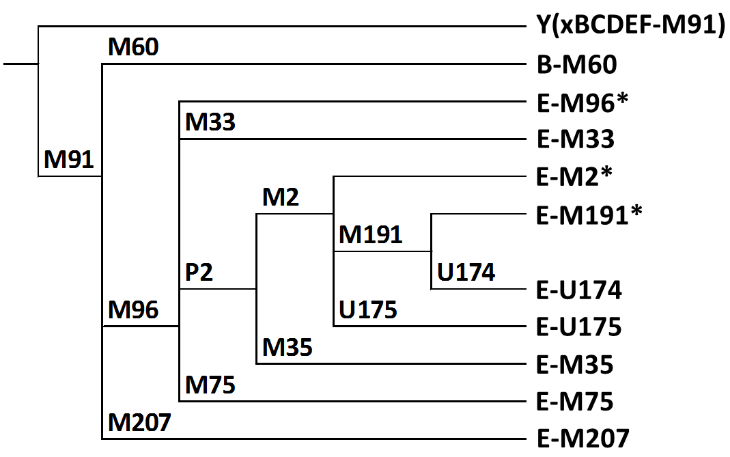

Supplement: S4 Fig — The nomenclature of the subhaplogroups is based on the terminal mutation that defines them, as proposed in van Oven et al. [40]. *Paragroups: Y-chromosomes not defined by any downstream-examined mutation. (TIF) [file pone.0141510.s004.tif]

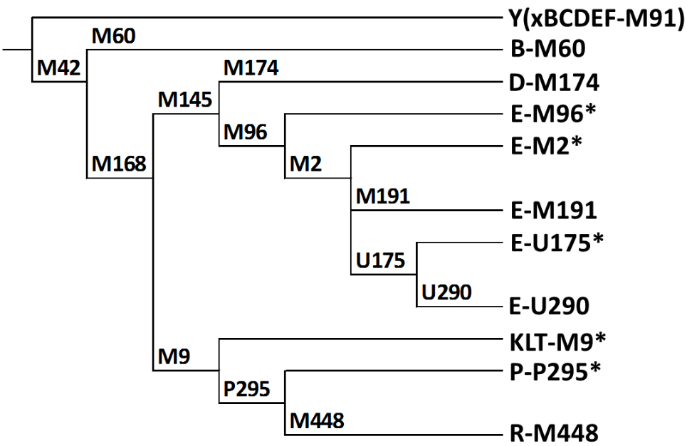

Supplement: S5 Fig — The nomenclature of the subhaplogroups is based on the terminal mutation that defines them, as proposed in van Oven et al. [40]. *Paragroups: Y-chromosomes not defined by any downstream-examined mutation. (TIF) [file pone.0141510.s005.tif]

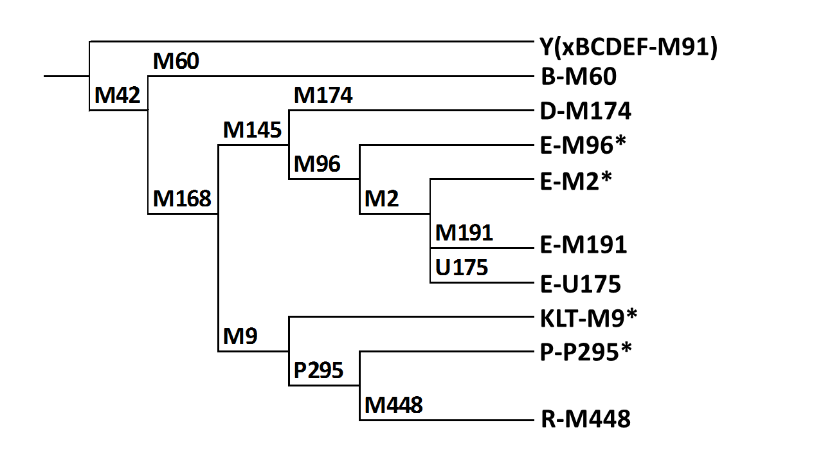

Supplement: S6 Fig — The nomenclature of the subhaplogroups is based on the terminal mutation that defines them, as proposed in van Oven et al. [40]. *Paragroups: Y-chromosomes not defined by any downstream-examined mutation. (TIF) [file pone.0141510.s006.tif]

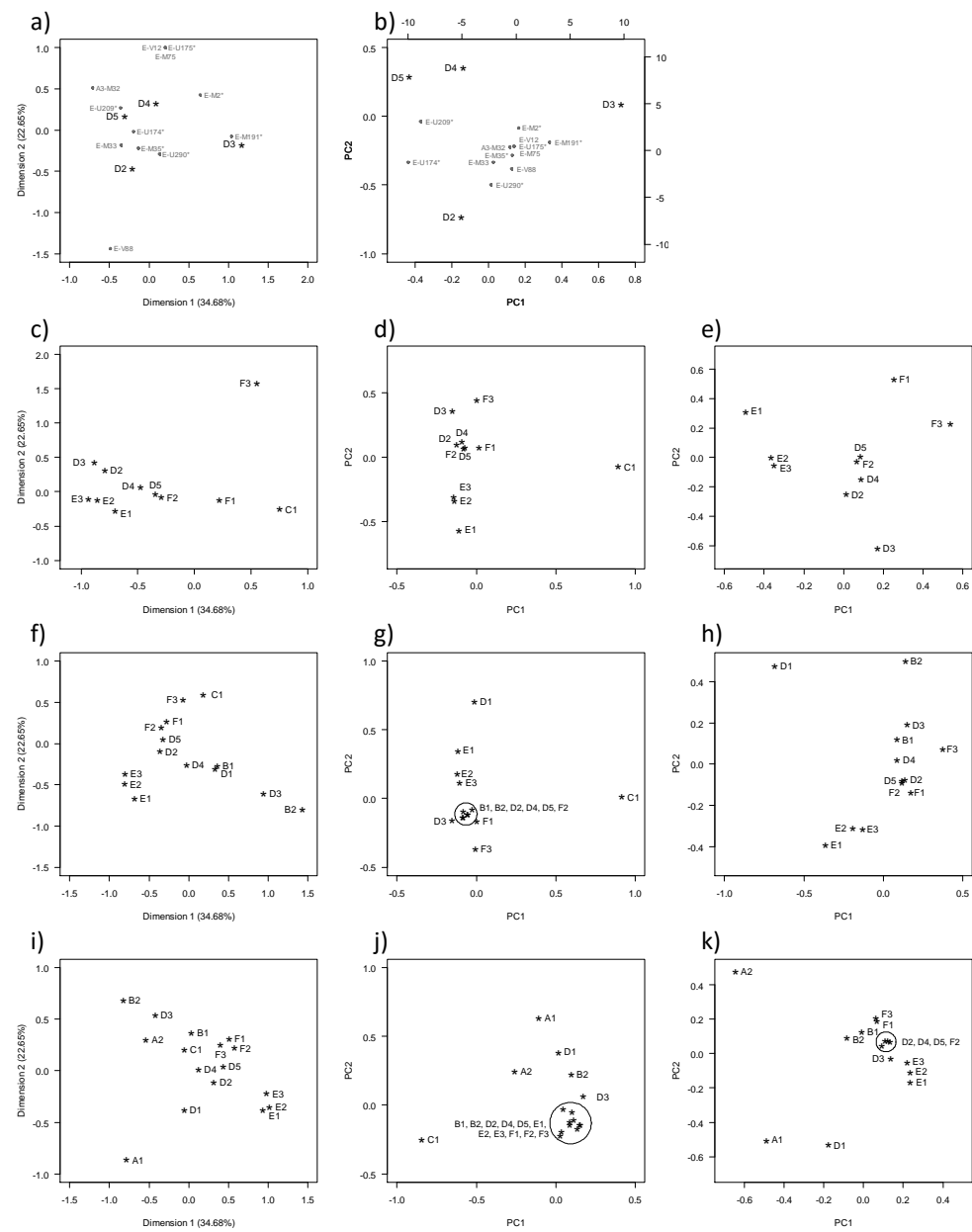

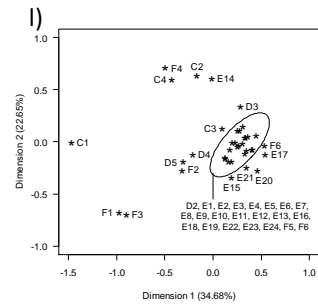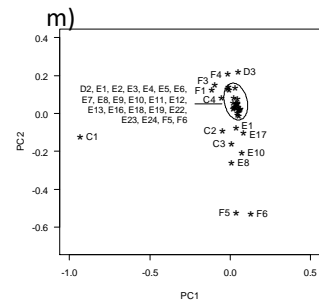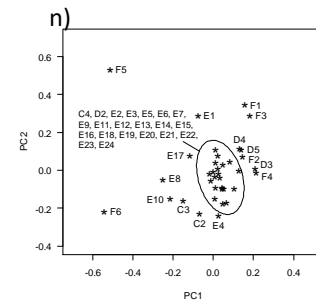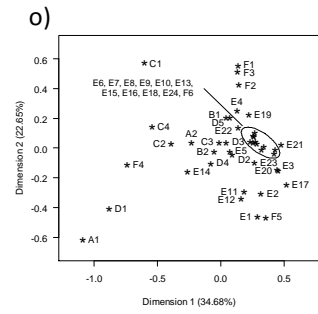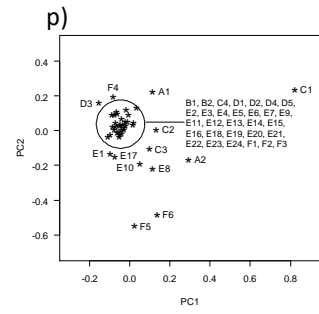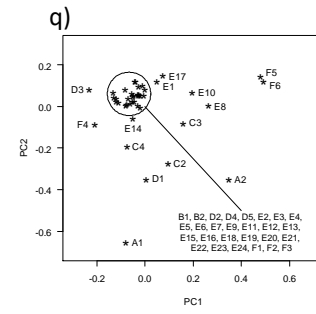

Supplement: S7 Fig — The nomenclature and the references of the population samples are available in S1 Table. With a) CA and b) PCA, together with a biplot, of the four–in this study re-genotyped–Beninese population samples based on the Y-SNP frequencies using the phylogeny given in S1 Fig. The cumulative proportion is 0.96 for the first two principal components (PC1: 0.82; PC2:0.14). With c) CA and d-e) PCA of the eleven West-African population samples with (d) and without (e) sample C1, all based on the Y-SNP frequencies using the phylogeny given in S2 Fig. The cumulative proportion of plot (d) is 0.98 for the first two principal components (PC1: 0.92; PC2: 0.06), and of plot (e) is 0.92 for the first two principal components (PC1: 0.64; PC2: 0.28). With f) CA and g-h) PCA of the 14 West-African population with (g) and without (h) sample C1, all based on the Y-SNP frequencies using the phylogeny given in S3 Fig. The cumulative proportion of plot (g) is 0.92 for the first two principal components (PC1: 0.83; PC2: 0.09), and of plot (h) is 0.81 for the first two principal components (PC1: 0.45; PC2: 0.36). With i) CA and j-k) PCA of 16 West-African population samples with (j) and without (k) sample C1, all based on the Y-SNP frequencies using the phylogeny given in S4 Fig. The cumulative proportion of plot (j) is 0.87 for the first two principal components (PC1: 0.74; PC2: 0.13), and of plot (k) is 0.79 for the first two principal components (PC1: 0.54; PC2: 0.25). With l) CA and m-n) PCA of 38 West-African population samples with (m) and without (n) sample C1, all based on the Y-SNP frequencies using the phylogeny given in S5 Fig. The cumulative proportion of plot (m) is 0.86 for the first two principal components (PC1: 0.58; PC2: 0.30), and of plot (n) is 0.83 for the first two principal components (PC1: 0.70; PC2: 0.13). With o) CA and p-q) PCA of the 43 West-African population samples with (g) and without (h) sample C1, all based on the Y-SNP frequencies using the phylogeny given in [file pone.0141510.s007.pdf]

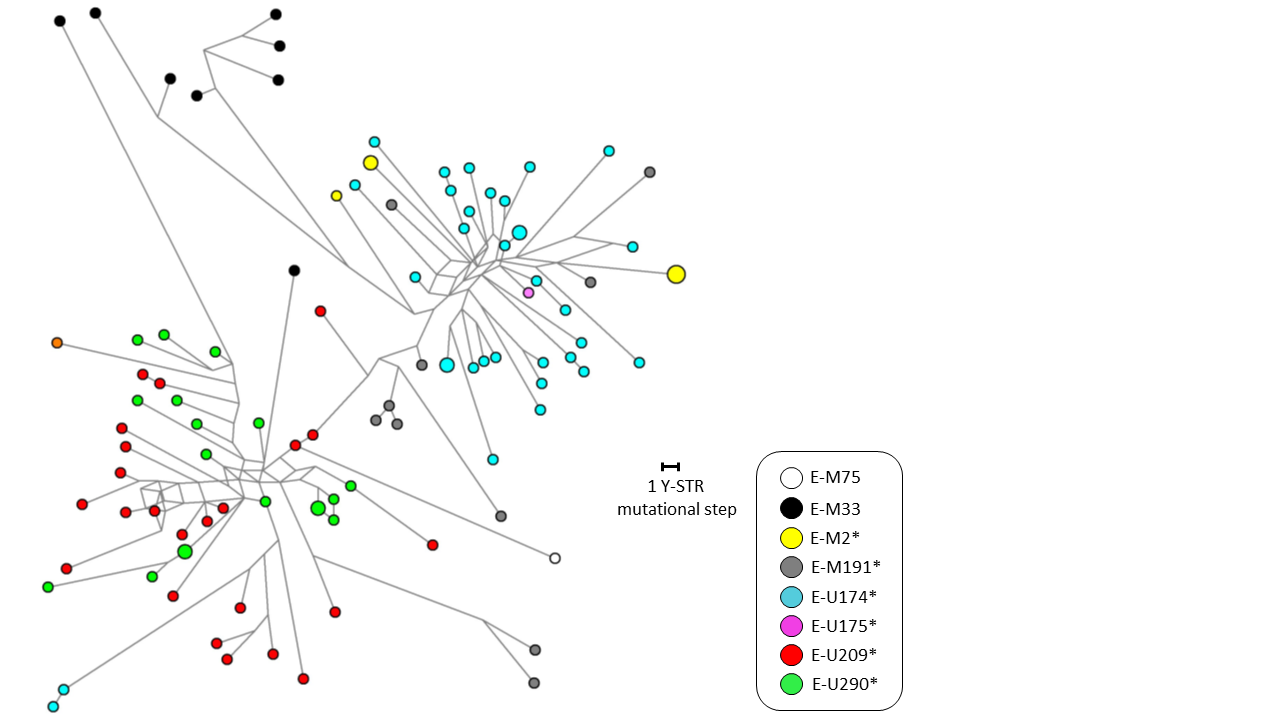

Supplement: S8 Fig — The size of the circles are proportional to the haplotype frequency. The colour of the circles represents the subhaplogroup to which the haplotype belongs based on Y-SNP typing and based on the phylogeny given in S1 Fig. (TIF) [file pone.0141510.s008.tif]

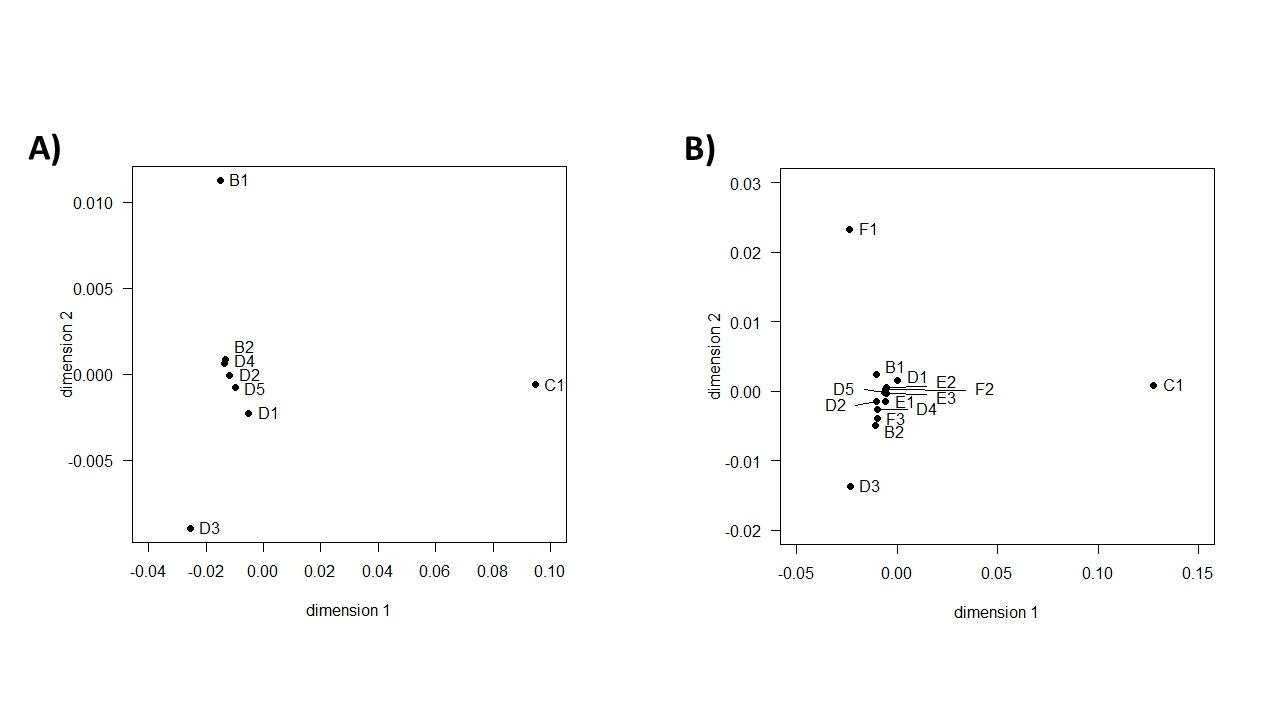

Supplement: S9 Fig — The nomenclature and the references of the population samples are available in S1 Table. (TIF) [file pone.0141510.s009.tif]

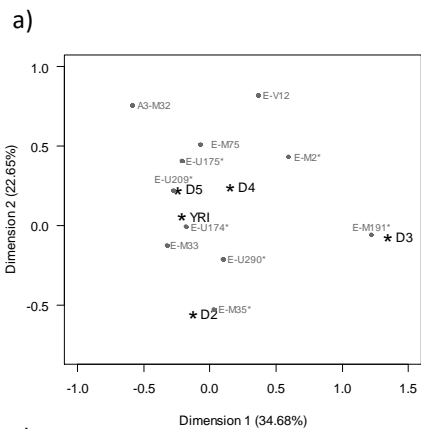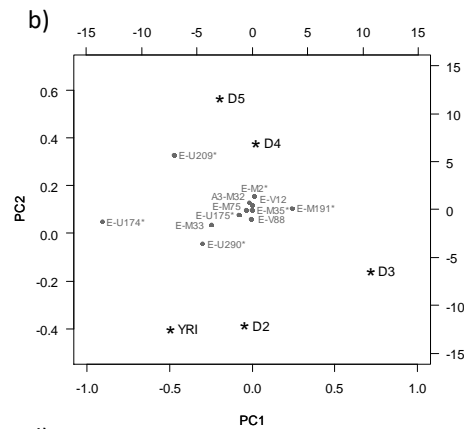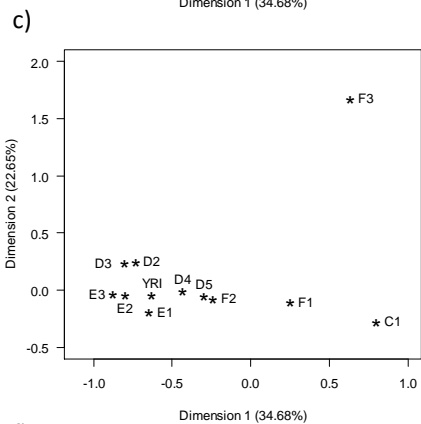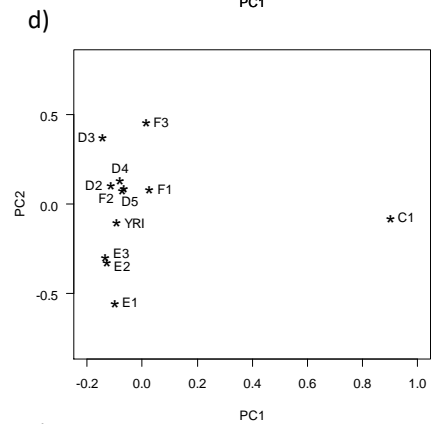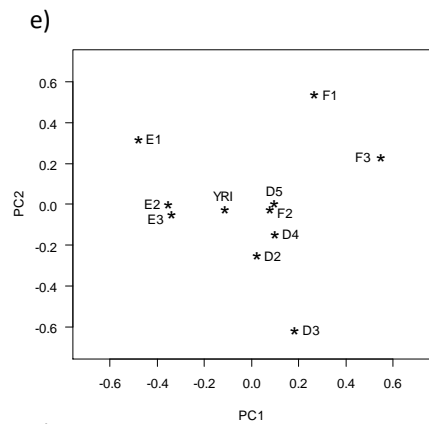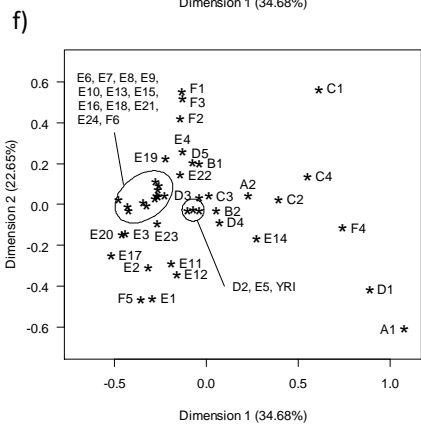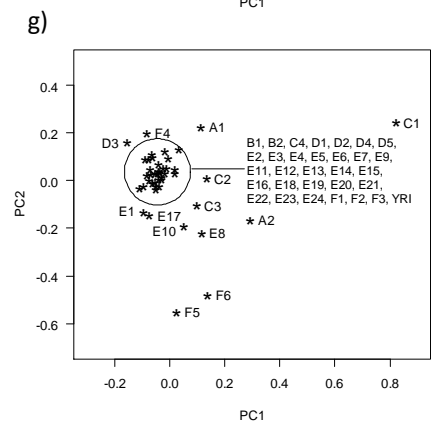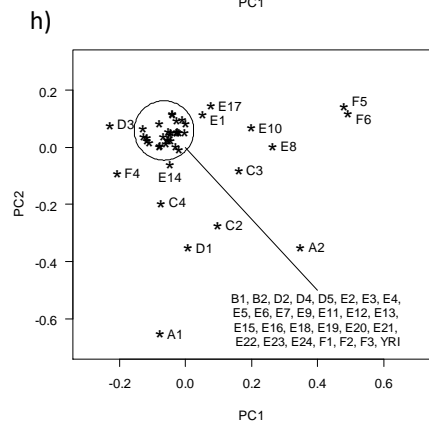

Supplement: S10 Fig — The nomenclature and the references of the population samples are available in S1 Table. With a) CA and b) PCA, together with a biplot, of YRI and the four–in this study re-genotyped–Beninese population samples based on the Y-SNP frequencies using the phylogeny given in S1 Fig. The cumulative proportion is 0.95 for the first two principal components (PC1: 0.80; PC2:0.15). With c) CA and d-e) PCA of YRI and the eleven West-African population samples with (d) and without (e) sample C1, all based on the Y-SNP frequencies using the phylogeny given in S2 Fig. The cumulative proportion of plot (d) is 0.98 for the first two principal components (PC1: 0.92; PC2: 0.06) and of plot (e) is 0.90 for the first two principal components (PC1: 0.63; PC2: 0.27). With f) CA and g-h) PCA of YRI and 43 West-African population samples with (g) and without (h) sample C1, using the phylogeny given in S6 Fig. The cumulative proportion of plot (g) is 0.86 for the first two principal components (PC1: 0.58; PC2: 0.28), and of plot (h) is 0.81 for the first two principal components (PC1: 0.55; PC2: 0.26). (PDF) [file pone.0141510.s010.pdf]

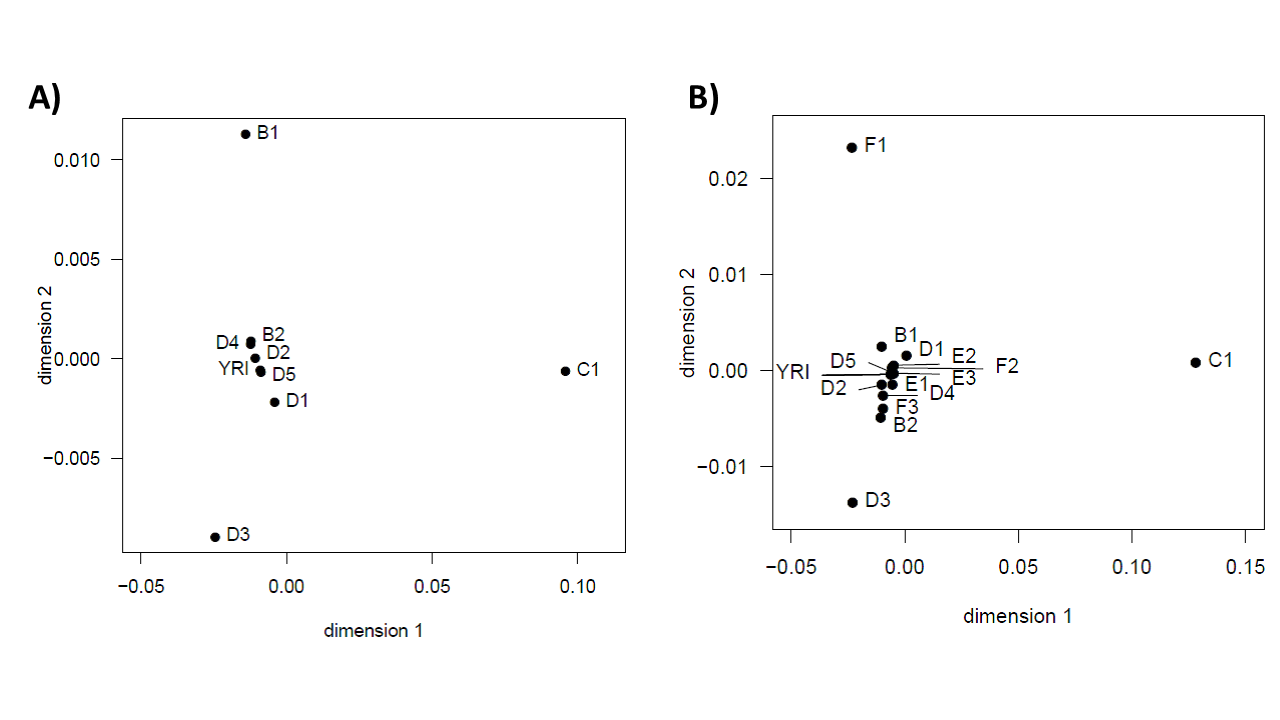

Supplement: S11 Fig — The nomenclature and the references of the population samples are available in S1 Table. (TIF) [file pone.0141510.s011.tif]
